# Diffuse arterial embolization secondary to pulmonary vein thrombosis

**DOI:** 10.1016/j.rmcr.2022.101732

**Published:** 2022-08-29

**Authors:** Keegan Plowman, Carl Ruthman

**Affiliations:** a Graduate Medical Education Internal Medicine Residency, NCH Healthcare System, 311 9th Street North, Naples, FL, 34102, USA; b Division of Pulmonary Critical Care Medicine, NCH Healthcare System, 311 9th Street North, Naples, FL, 34102, USA

**Keywords:** Pulmonary vein thrombosis, Pulmonary malignancy

## Abstract

Pulmonary vein thromboses (PVT) is a complication of lung malignancy and can be complicated by arterial embolic phenomenon. Patient is a 69-year-old female with a history of coronary artery disease and recent pneumonia seen on chest x-ray who presented with progressive headache, left arm numbness and abdominal pain. She was found to have numerous bilateral strokes, bilateral renal infarction and splenic infarction. CT Chest showed a right upper lobe mass compressing the pulmonary veins with filling defects concerning for thrombus. Diagnosis of PVT is made with CT angiogram, echocardiogram and MRI. Treatment consists of anticoagulation and treatment of underlying factors.

## 1 Introduction

The pulmonary veins transport oxygenated blood from the pulmonary circulation into the left side of the heart and subsequently into systemic arterial circulation. Pulmonary vein thromboses (PVT) is a described complication of lung malignancy [1], lung resections [2], lung transplantation [3], thoracic surgical intervention [2], radiofrequency ablation treatment for atrial fibrillation [4], polycythemia vera [5], COVID [6] or can even develop spontaneously [7]. The development of pulmonary vein thrombosis (PVT) can be complicated by clot dislodgement into the arterial circulation and subsequent arterial systemic embolic phenomenon with infarcts visualized in numerous organs. There are reported cases of ischemic stroke, renal infarction [8] and diffuse alveolar hemorrhage [9] secondary to PVT. These can contribute to significant clinical morbidity and mortality for patients.

## 2 Case report

Patient is a 69-year-old female with history of coronary artery disease, ulcerative colitis, hypothyroidism, hyperlipidemia and recent pneumonia seen on chest x-ray who presented with progressively worsening headache, transient left arm numbness and abdominal pain. CT head was negative for acute hemorrhage. National Institute of Health Stroke Scale was 1. Neurology was consulted who recommended MRI brain. MRI showed multifocal acute and subacute infarcts in the supratentorial and infratentorial brain; in the bilateral frontal parietal cortical regions; occipital temporal regions; right thalamus and bilateral cerebellum. CT abdomen and pelvis showed bilateral renal infarctions and numerous splenic infarctions. The morning after admission the patient developed acute onset facial droop and slurred speech. CT head showed subacute right middle cerebral artery (MCA) and bilateral posterior cerebral artery (PCA) infarcts without hemorrhage, herniation or mass-effect/midline shift. CT angiogram of the neck was negative for any acute narrowing or aneurysm. CT perfusion showed a moderate sized area of ischemia in the left occipital and right temporal occipital lobes with large surrounding penumbra. CT angiogram of the head showed interval development of two noncalcified, soft thrombi located in the right MCA bifurcation and left MCA. Patient was taken for mechanical thrombectomy of the left MCA with good angiographic results with Thrombolysis in Cerebral Infarction score of 2c. Patient had worsening neurological exam overnight with increased somnolence which prompted repeat imaging. CT head overnight showed subacute right MCA/PCA, left MCA and left cerebellar infarctions, increasing edema with a 2 mm midline shift, no signs of herniation and subarachnoid hemorrhage. Transthoracic echocardiogram was negative for left atrial or left ventricular thrombus. CT Chest showed large right upper lobe mass with associated mediastinal and right hilar lymphadenopathy. The mass was compressing the pulmonary veins with filling defects concerning for thrombus. Due to the extensive stroke burden the patient was unable to receive systemic anticoagulation and the patient was transitioned to comfort measures. Transesophageal echocardiogram, biopsy of the mass and thrombophilia workup were deferred because of the family's decision to transition to hospice care.

## 3 Discussion

PVT can be an unfortunate complication of malignancy, especially pulmonary malignancy. The case demonstrates a large right upper lobe lung mass compressing the pulmonary veins. External compression likely caused stasis and coupled with a hypercoagulable state in the setting of malignancy led to the development of pulmonary vein thrombosis. Subsequent thromboembolic phenomena led to diffuse infarction in multiple organ systems. Due to the acuity of the patient's symptoms and clinical deterioration, the gold standard imaging tests were unable to be obtained. Diagnosis can be challenging but detection of PVT is increasing with improvements in imaging technique. Typically, a combination of imaging techniques are used to best diagnose PVT. CT scan with IV contrast with timing for the pulmonary venous system is the best diagnostic technique [3]. Transesophageal echocardiogram can best visualize left atrial thrombus that are often missed on transthoracic echocardiogram [1]. More recently MRI/MRV have been used to distinguish between filling defects and tumor invasion [10]. PVT can have devastating consequences when diffuse embolization occurs. PVT is an uncommon condition and there are no established guidelines for treatment. Therapy generally focuses on treating the underlying cause. Lung resection is often performed to treat malignancy or external compression [3]. Anticoagulation is initiated unless contraindicated [7]. There are case reports of resolution of thrombus with the use of dabigatran [11] and warfarin [7]. Antibiotics are generally given prophylactically [3]. Thrombectomy has been used for acute management after lung transplant complicated by pulmonary vein thrombosis [12]. Percutaneous angiovac catheter based thrombectomy is a novel alternative to conventional surgical thrombectomy for intravascular or intracardiac thrombus [13]. This is an alternative that needs to be explored due to the high risk for significant deficits due to systemic infarctions. There should be high clinical suspicion for PVT in patients with diffuse arterial system emboli and risk factors for thrombus formation.

## Learning points

- Patients with diffuse arterial emboli should be evaluated for pulmonary vein thrombosis, left atrial thrombus, left ventricular thrombus and systemic shunts
- Workup includes CT imaging with timing of contrast to evaluate the pulmonary veins, MRI/MR venogram, transthoracic echocardiogram and transesophageal echocardiogram
- Systemic anticoagulation should be initiated when diagnosis is suspected to avoid diffuse arterial embolization

## Sources of funding

This work was performed as part of employment under Naples Community Hospital Healthcare System. The authors listed above are all employed within this system.

## Declaration of competing interest

The authors listed above certify that they have no financial or non-financial interest in the subject matter or materials discussed in this manuscript.
