# Supplementary material for: Diffuse arterial embolization secondary to pulmonary vein thrombosis
Source: Respir Med Case Rep. 2022 Aug 29;39:101732. doi: 10.1016/j.rmcr.2022.101732 (PMC9465264; doi:10.1016/j.rmcr.2022.101732)
Supplement: Multimedia component 1 [file mmc1.docx]

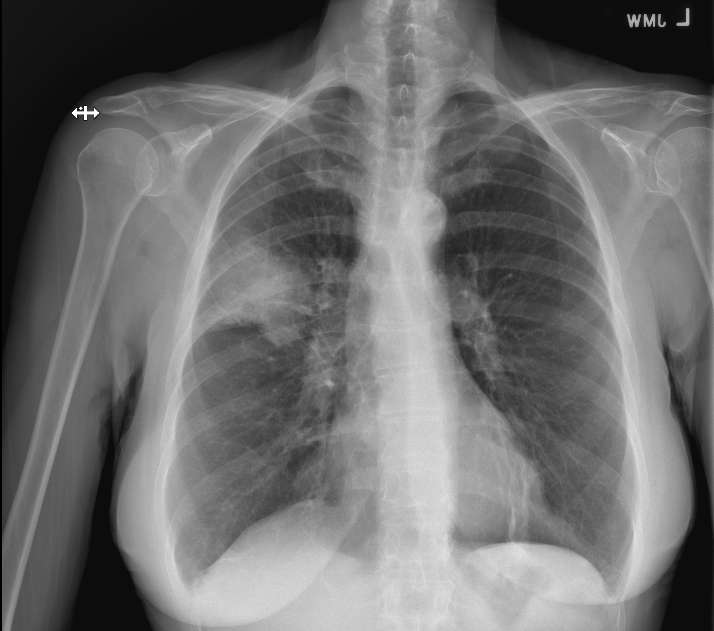

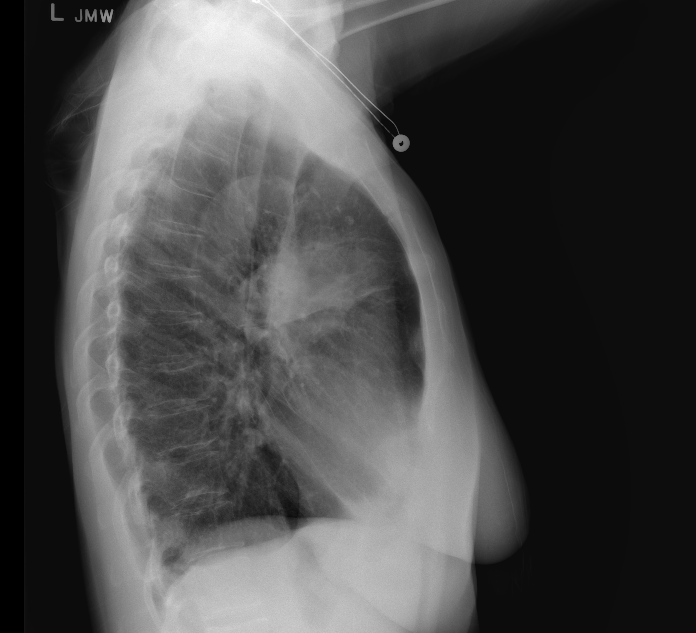


**Figure 1:** CXR PA/Lateral showing right upper lobe opacity


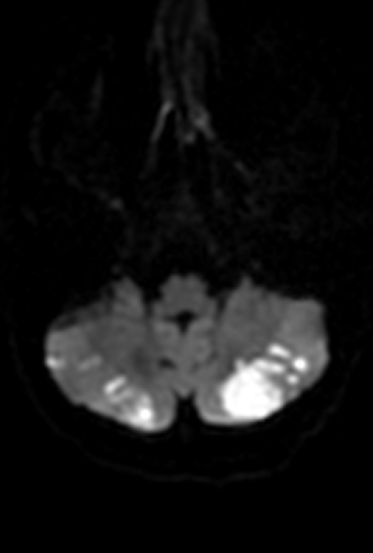

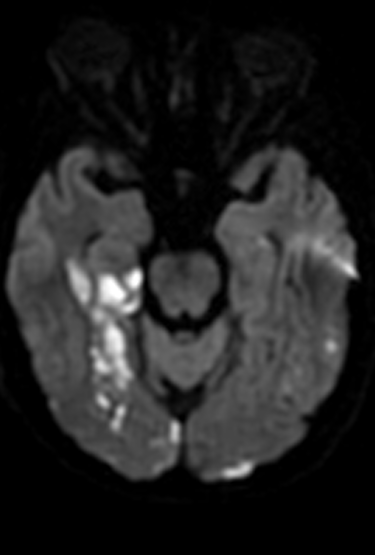

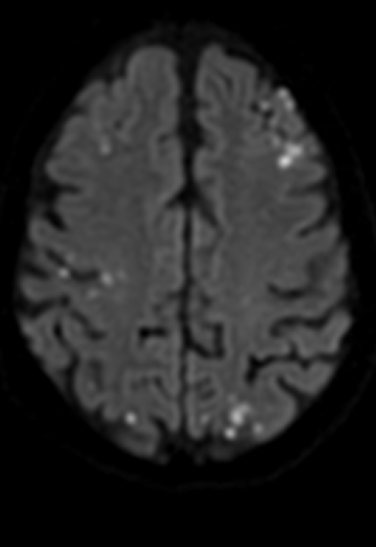


**Figure 2:** MRI brain showing numerous bilateral strokes


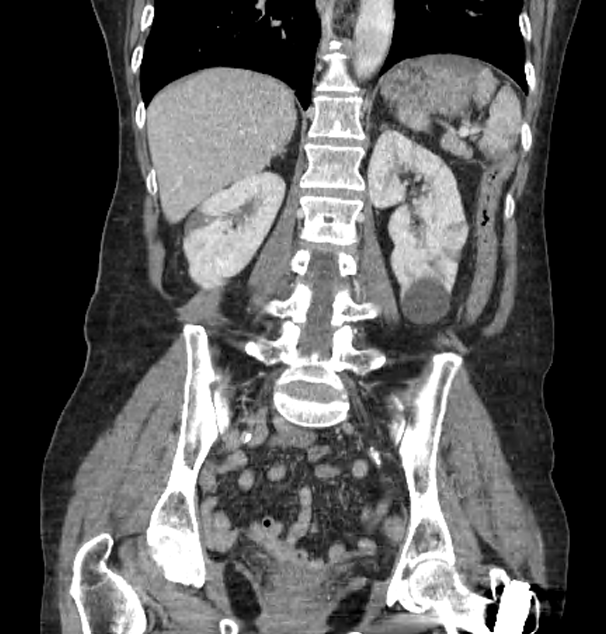

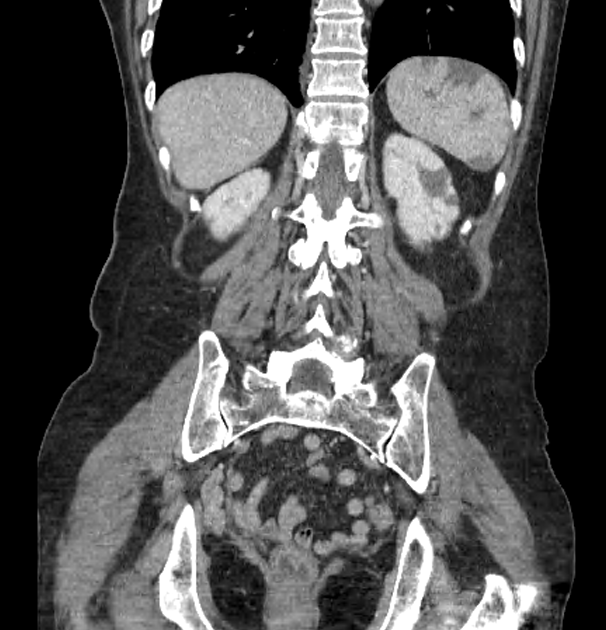


**Figure 3:** CT Abdomen pelvis showing numerous bilateral renal and splenic infarcts


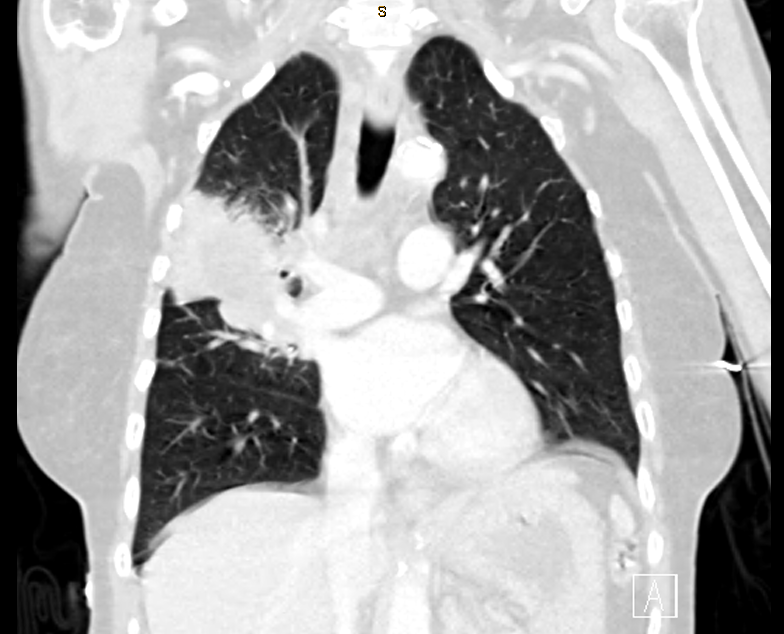

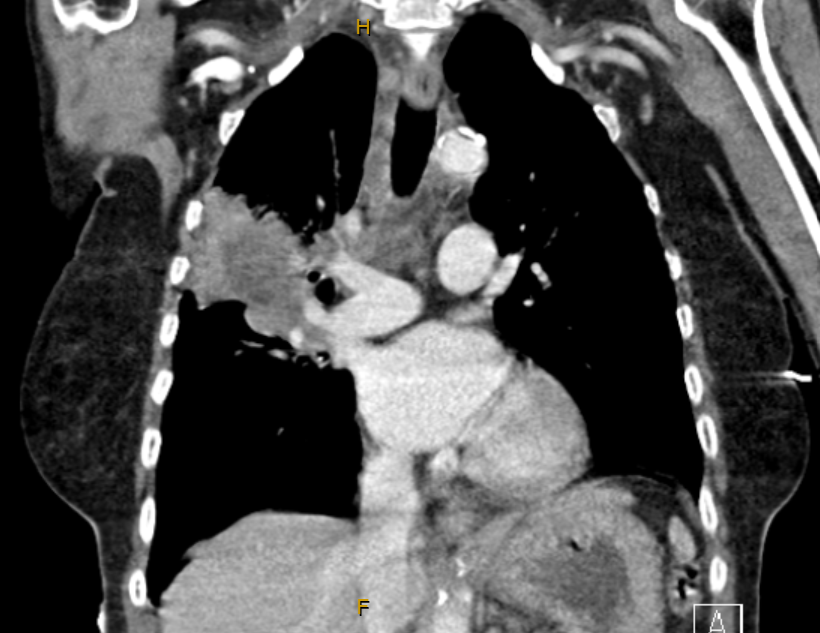


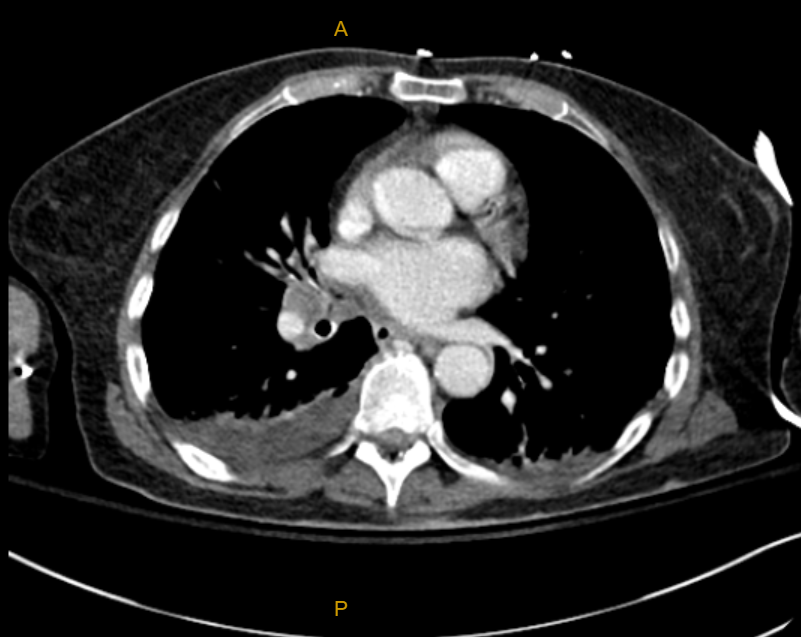

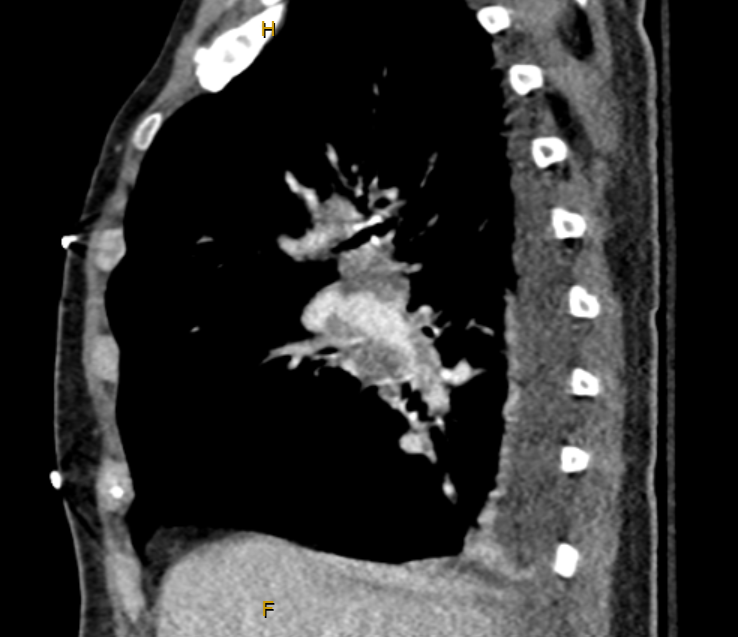


**Figure 4:** CT Chest showing right upper lobe mass compressing the pulmonary veins with filling defects concerning for thrombus
